# Helium ion beam imaging for image guided ion radiotherapy

**DOI:** 10.1186/s13014-018-1046-6

**Published:** 2018-06-14

**Authors:** M. Martišíková, T. Gehrke, S. Berke, G. Aricò, O. Jäkel

**Affiliations:** 1 Department of Radiation Oncology and Radiation Therapy, Heidelberg University Hospital, Im Neuenheimer Feld 400, 69120 Heidelberg, Germany; 2 Medical Physics in Radiation Oncology, German Cancer Research Center (DKFZ), Im Neuenheimer Feld 280, 69120 Heidelberg, Germany; 3 Heidelberg Institute for Radiation Oncology (HIRO), National Center for Radiation Research in Oncology, Im Neuenheimer Feld 400, Heidelberg, Germany; 4 Heidelberg Ion Beam Therapy Center, Im Neuenheimer Feld 450, 69120 Heidelberg, Germany; 5 Present address: The Royal Liverpool and Broadgreen University Hospitals NHS Trust, Prescot Street, Liverpool, L7 8XP UK; 6 Present address: European Organization for Nuclear Research CERN, CH-1211 Geneva 23, Switzerland

**Keywords:** Ion beam radiotherapy, Image guidance, Ion radiography, Helium ion beams, Pixelated semiconductor detector Timepix

## Abstract

**Background:**

Ion beam radiotherapy provides potential for increased dose conformation to the target volume. To translate it into a clinical advantage, it is necessary to guarantee a precise alignment of the actual internal patient geometry with the treatment beam. This is in particular challenging for inter- and intrafractional variations, including movement. Ion beams have the potential for a high sensitivity imaging of the patient geometry. However, the research on suitable imaging methods is not conclusive yet. Here we summarize the research activities within the “Clinical research group heavy ion therapy” funded by the DFG (KFO214). Our aim was to develop a method for the visualization of a 1 mm thickness difference with a spatial resolution of about 1 mm at clinically applicable doses.

**Methods:**

We designed and built a dedicated system prototype for ion radiography using exclusively the pixelated semiconductor technology Timepix developed at CERN. Helium ions were chosen as imaging radiation due to their decreased scattering in comparison to protons, and lower damaging potential compared to carbon ions. The data acquisition procedure and a dedicated information processing algorithm were established. The performance of the method was evaluated at the ion beam therapy facility HIT in Germany with geometrical phantoms. The quality of the images was quantified by contrast-to-noise ratio (CNR) and spatial resolution (SR) considering the imaging dose.

**Results:**

Using the unique method for single ion identification, degradation of the images due to the inherent contamination of the outgoing beam with light secondary fragments (hydrogen) was avoided. We demonstrated experimentally that the developed data processing increases the CNR by 350%. Consideration of the measured ion track directions improved the SR by 150%. Compared to proton radiographs at the same dose, helium radiographs exhibited 50% higher SR (0.56 ± 0.04lp/mm vs. 0.37 ± 0.02lp/mm) at a comparable CNR in the middle of the phantom. The clear visualization of the aimed inhomogeneity at a diagnostic dose level demonstrates a resolution of 0.1 g/cm^2^ or 0.6% in terms of water-equivalent thickness.

**Conclusions:**

We developed a dedicated method for helium ion radiography, based exclusively on pixelated semiconductor detectors. The achievement of a clinically desired image quality in simple phantoms at diagnostic dose levels was demonstrated experimentally.

## Background

The delivery of a sufficient dose to control the tumor growth can be challenging when an organ-at-risk (OAR) is close to the target, or for radioresistant tumors which require high tumor doses. The physical and radiobiological properties of the therapeutic ion beams allow for an increased dose conformation to the tumor in comparison to standard radiotherapy with photon beams (e.g. [55]). The superior dose distributions theoretically achievable with ions can be compromised by uncertainties from different sources in the clinical practice. From the geometrical point of view, interfractional changes along the course of the radiotherapy might arise due to patient positioning uncertainties, anatomical changes including weight gain or loss, or swelling of tissue. In addition, intrafractional changes might occur as a result of movement on different time scales: muscles (minutes to seconds), breathing (seconds), and heart beat (below seconds). Moreover, the conversion of the attenuation data acquired by a CT to the stopping power distribution, which is needed as an input to the treatment planning, is associated with range uncertainties of 2-3% and in some cases even more [29, 30, 34].

The uncertainties from all the known sources translate into the size of margins around the target, which are designed to assure that the tumor receives the planned dose. However, the larger the margins, the higher is the radiation exposure of the surrounding healthy tissues. This might lead to an increased rate and severity of side effects, leading to the limitation of the maximal dose which can be realistically applied to the tumor.

### Imaging in ion beam radiotherapy

Due to the reasons given above, image guidance has a potentially higher impact on the quality of the delivered dose distribution in ion radiotherapy than it is the case in photon radiotherapy. However, in clinical practice dedicated imaging techniques are currently less deployed in ion radiotherapy than in photon radiotherapy [60]. This contrasts with the high complexity of the ion beam radiotherapy centers.

Markerless imaging techniques provide advantages in terms of their potential precision and no need for the invasive for marker implantation. The potential of on-couch X-ray imaging used for intrafractional monitoring of the anatomical changes is limited due to the inherent poor soft tissue contrast. Moreover, the desired information about the actual stopping position of the ion beam in the patient cannot be obtained directly. The use of dual-energy CT for an improved stopping power determination [69] can be potentially in-room, but it is not suited for an in-beam imaging of moving organs. An in-beam MR imaging would provide the advantage of a high soft tissue contrast without any additional dose to the patient. The development of this technique is at its very beginning [47]. In particular, the determination of the stopping power with the required precision from the MR images is not solved yet.

Patient imaging techniques specific to ion radiotherapy are represented essentially by in-vivo monitoring techniques and by ion radiography, which have the potential to uncover the actual stopping power discrepancies in the treatment position. The main in-vivo treatment monitoring techniques under development are based on the detection of secondary radiation arising as a consequence of nuclear reactions of the treatment beam with the nuclei of the patient’s tissue [37]. Despite the demonstrations of the clinical feasibility for some of them ([40, 53], and references within), the techniques are currently still under development and evaluation, and none of them is clinically widespread yet.

### Transmission imaging with ions

The motivation for ion based imaging^1^ in the field of ion radiotherapy is twofold. Firstly, an acquirement of quantitative anatomic information on the stopping power distribution of the patient in the treatment position within the coordinate system of the treatment room is conceivable. Secondly, due to the potentially achievable high contrast, ion based imaging is a candidate for an on-couch detection of the tumor position within the patient anatomy. For the majority of treatment sites, this kind of imaging could be performed in the treatment position in beam’s-eye-view.

Transmission imaging with ion beams takes advantage primarily from the distinct shape of the Bragg-curve. In comparison to the attenuation curve of photons, which is exploited for X-ray imaging, the range of therapeutic ions in tissue is finite. Therefore, when considering a known initial energy of a monoenergetic primary ion beam, the measured residual energy (or range) of the transmitted ions behind the imaged object provides a direct information about the stopping power of the object. Moreover, the Bragg peak exhibits a steep rise on both proximal and distal flanks. Measuring the energy loss of the beam in this region enables to reach a high sensitivity to small areal-density changes along the beam path in the imaged object. Further potentially usable contrasts include attenuation of the ion fluence and nuclear scattering [62].

The key element of the majority of the published radiographic systems is the detector sensitive to the residual energy of the beam (or single ions), which is emerging from the imaged object. Besides the direct measurement of the residual energy by a calorimeter, residual range telescopes are often used [51]. They exploit the fact that the residual range is monotonic with the residual energy. For the measurement of both the residual energy or range, detectors are needed which are thick enough to stop the entire beam within its sensitive volume. Other approaches are based on the measurement of the energy loss within thin detectors situated in the rising part of the Bragg peak [36]. In this region the energy loss is also monotonically dependent on the outgoing ion energy, albeit in a narrow range of water equivalent thickness (WET) of the imaged object.

In general, the attainable spatial resolution is limited by the multiple Coulomb scattering of the imaging beam within the imaged object. The lighter the ion, the broader is the lateral spread of the beam behind a given thickness of the traversed material [55]. Therefore, tracking detectors are implemented in most radiographic systems, in particular for proton based imaging, in order to measure the positions or even directions of single ions in front and/or behind the imaged object [33, 51]. The knowledge of the directions of the incoming and outgoing ions enables to increase the spatial resolution of the images by calculation of the most probable paths within the imaged object [15, 23, 58, 68].

### Status of the research

#### Radiation detection systems for ion imaging

Imaging with ions of sufficient energy to cross a patient-relevant WET is bound on high energy ion beam facilities. The first published proton radiography [35] stands at the beginning of a series of pioneering publications on proton imaging research initiated at Harvard, Cambridge, MA, USA. The image contrast was based on the attenuation of the energy fluence of the proton beams. The initially used passive 2D solid state detectors [62] were replaced from mid 70’s by active integrating electronic detection systems using collimators [12, 38, 43].

The research at the Lawrence Berkeley Laboratory, CA, USA was focused on imaging with heavier ions, mainly helium, carbon and oxygen. Within this project, the first electronic particle scanner was developed and investigated for helium ion imaging [13]. It comprised a scintillation based range counter complemented by a tracker based on multiwire proportional chambers (MWPCs). The concept of the detection system – composed of a particle tracker and a calorimeter - is today the mostly exploited method for ion radiography.

The proton CT technique developed at the Los Alamos National Laboratory, NM, USA, used a residual range telescope made of plastic scintillation slabs. The tracker was composed of position sensitive proportional chambers [23, 24]. In the early 80’s this *first era of ion transmission tomography* got to its end. Its driving motivation had been the diagnostic imaging with increased WET-contrast in comparison to X-rays [62] and a possibly reduced imaging dose to the patient.

The so-called *modern era of ion transmission tomography* [51] started with the developments at the Paul Scherrer Institute (PSI) in Switzerland in mid 90’s. Today the cost/performance of X-ray and MR-imaging in diagnostics is hard to compete with. However, with the spread and commercialization of the ion beam radiotherapy, there is a need for an on-couch tumor visualization and an accurate determination of the stopping power of patient tissue for planning of ion radiotherapy. Moreover, clinically usable methods for stopping power verification, ideally in-vivo, are also desired.

Despite the five decades which passed since the first published ion radiograph, the question of the optimal choice of the detection system components and the measurement method is not finally answered yet. The research is governed by the following considerations: for a realistic clinical application, the maximal time span between the beginning of the imaging and the time of the image availability has to be below 10 min for interfractional on-couch imaging directly before the treatment, and even shorter for intrafractional imaging. This automatically rules out passive radiation detectors. The potentially usable electronic detectors can be divided into integrating and single particle detectors. Up to now the vast majority of ion imaging systems was designed for proton imaging. Due to their significant scattering in comparison to heavier ions, single particle detection is the method of choice when clinically relevant spatial resolution is aimed for. On the contrary, integrating detectors are not capable to resolve single particles. Hence, their use is basically limited to heavy ions, which exhibit a decreased lateral scattering in the patient [1, 54, 59, 64, 66, 70].

Since we aimed to build a system that is potentially capable to operate with different ion types including protons, integrating detectors were not suitable. Therefore, in the following we focus on single particle systems only. The majority of the developed trackers are based on solid state technologies like scintillation fibers [36, 45, 46, 57] and silicon strip detectors [32, 63].

Systems for measurement of the residual energy include calorimeters based on crystals [10, 28] or plastic scintillators [3, 45]. Alternative range telescopes are mainly composed of scintillation slabs [2, 46, 57]. A special category are tracking calorimeters based on scintillating blocks [9] and scintillating fiber arrays for energy loss measurements [36]. The exploitation of pixelated semiconductor detectors for building of ion radiographic systems is currently at its very beginning [48, 52].

#### Ion species for imaging

Due to their widest availability, protons are up to now the most investigated ion type for radiography [33]. However, the limitation of the proton imaging by multiple Coulomb scattering within the imaged object was documented in several works (e.g. [49, 68, 70]).

The implementation of heavy ions (carbon or oxygen) imaging into clinics might pose a problem due to their high linear energy transfer (LET) in comparison to protons. While short term side effects can be included in the biological dose estimation, long term effects of high LET radiation are not sufficiently investigated yet. Moreover, the dose per particle is significantly higher than for lighter ions.

Helium ions, which have been up to now experimentally investigated for imaging in several works only [13, 45, 67], might represent the optimal ion imaging modality. Due to their increased mass with respect to protons, their lateral spread caused by multiple scattering is by a factor of 2 lower than for protons of the same range [27, 42]. Therefore, the spatial resolution is expected to be significantly improved in comparison to protons [21].

For imaging with ions heavier than protons, secondary fragments represent a challenge [4]. They do not have just different ranges, but also different spatial distributions, lower charges and thus lower energy depositions than the primary ions. Therefore, lighter secondary ions cause a decrease of the measured WET resolution.

## Methods

For a future identification of inter- and intra-fractional changes in the patient anatomy in the coordinate system of the treatment room, we have developed an imaging method based on ion radiography. To make a real clinical advancement, a sufficient contrast-to-noise ratio (CNR) enabling to distinguish a 1% difference in WET and a spatial resolution (SR) of about 1 mm [51] are needed. Acceptable integral patient doses and imaging times as well as fast data processing are a necessary prerequisite for the future clinical implementation.

Due to the shorter imaging time, lower technical complexity and the lower radiation dose, ion radiography is more straight forward to be implemented into clinics than ion tomographic imaging. Radiographic 2D imaging can be in principle performed directly before and after the treatment, as well as between the single delivered energy layers of the treatment plan. Therefore we focused in particular on ion radiography.

The residual energy or its surrogates (e.g. residual range) are the main radiographic quantities used for ion imaging. The quality of the images is typically quantified by two parameters: spatial resolution and contrast-to-noise ratio. SR reflects the ability to differentiate two regions with different WET in close vicinity to each other. SR in radiography is defined in the image plane, i.e. the plane perpendicular to the beam direction. CNR is a measure of the resolution of the object thickness in the beam direction. Our aim was to maximize both SR and CNR at clinically acceptable imaging doses. These criteria guided us in the choice of the imaging beam type, the detection technology, design of the radiographic detection system and in the development of the dedicated data postprocessing method [20, 21].

### Helium ion radiography

The experimental part of the study was performed at the Heidelberg Ion-Beam Therapy Center (HIT) [11] in Germany. HIT provides clinical treatments with proton and carbon ion beams since 2009, while helium ion treatments are going to start in close future. The beam delivery exploits dynamically modulated ion beams using the technology of active energy variation together with lateral pencil beam scanning [22]. The HIT synchrotron is capable to deliver ion beams with energies exhibiting ranges in water between 2 and 30 cm in 1-1.5 mm steps [11].

In our selection of the optimal imaging ion type we considered both physical and biological properties of the different ion types [21], as discussed in “Ion species for imaging” section. Helium was selected as the most promising modality to be evaluated. Therefore the method was developed specifically for helium ions. Consequently, one of the foci was to identify and exclude the background of light nuclear fragments (hydrogen) from the image [20]. Nevertheless, the usability of the system with other ion types was also an important criterion, in order to facilitate a systematic evaluation of the different ion imaging modalities with the same system [21].

### Pixelated semiconductor detector system for ion radiography

#### Radiation detection system

The presented method of helium radiography is based on a dedicated detection system designed for this purpose [20, 21]. In contrast to the majority of the ion radiographic / tomographic systems exploiting residual particle energy (or range), the main quantity measured here is the energy deposition in a thin layer (< 1 mm) on a single particle basis. Our method exploits the steep rise of the energy deposition closely before the Bragg peak to gain potentially higher image contrast.

In order to minimize the effect of image smearing due to multiple Coulomb scattering of the ions in the imaged object, the directions of both the incoming and outgoing ions are of interest. Therefore a tracking system composed of a front and a rear tracker was implemented in the design. This enabled us to measure single ion trajectories in front and behind the imaged object. To avoid deterioration of the images by hydrogen ions produced in nuclear interactions of primary helium ions within the imaged object, we integrated a unique method for ion identification based on pattern recognition of their signal [18, 26] into our imaging approach.

**Fig. 1:**
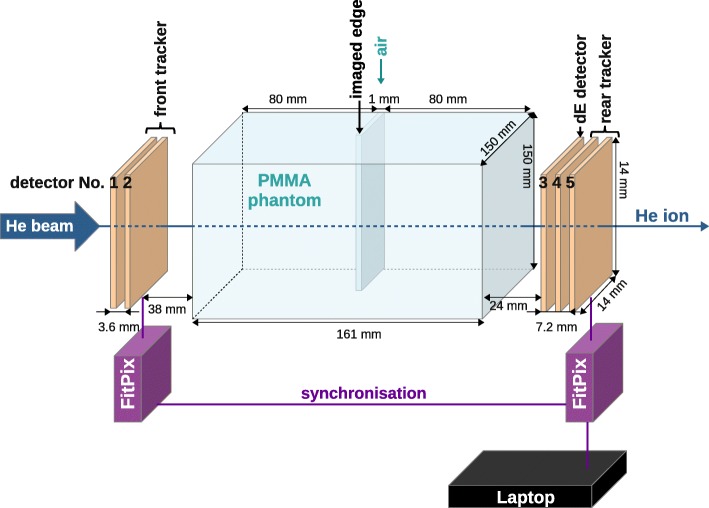
Experimental setup consisting of the front tracker, PMMA phantom with 1 mm air inhomogeneity, energy deposition detector and the rear tracker. FitPIX interfaces are used for data readout and synchronization. Data are saved to a laptop. Dimensions are not to scale. The PMMA phantom is 161 mm long, with the air inhomogeneity exactly in the middle. The pencil beam has energy of 168.3 MeV/u and width of 4.5 mm (FWHM)

In order to measure position and direction of single incoming and outgoing ions, energy deposition of the outgoing ions and type of the ion, the final version of the radiographic system consists of 5 sensitive layers of pixelated semiconductor detectors^2^ [21] as shown in Fig. 1. The parallel detector layers were arranged perpendicular to the beam axis. Two layers (detectors number 1 and 2) were used as a front tracker, two layers (detectors number 4 and 5) as a rear tracker. An additional layer (detector number 3) was used for the energy loss measurement and particle identification directly behind the imaged phantom (see Fig. 1).

#### The Timepix detectors

The chosen detection technology called Timepix was developed by the Medipix Collaboration at CERN [41]. We have opted for this technology because of its unique combination of several capabilities, which are beneficial for our purposes. Timepix enables a noise-free detection of single particles using a per-pixel adjustable threshold. Moreover, it exhibits detection efficiency close to 100% for heavy charged particles like the therapeutic ions down to protons. Besides the energy deposition information, the time-of-arrival can be measured. A synchronized operation of several layers measuring the arrival time and the coordinates of a particle’s impact enables single ion tracking [61]. An important criterion was also the straight forward manageability. The whole detection system is read out via USB and just a laptop is needed for its steering and data acquisition.

**Fig. 2:**
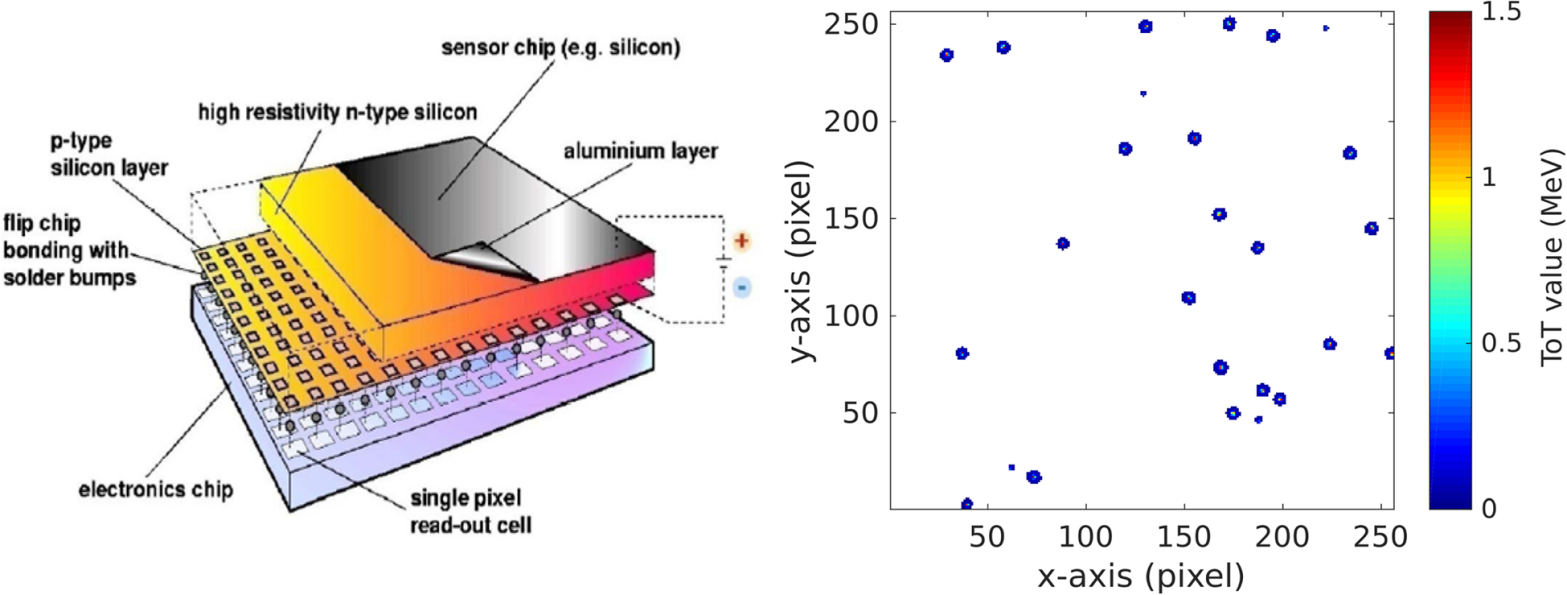
Left side: Visualization of a single Timepix detector structure by the Medipix Collaboration (www.cern.ch/medipix). Right side: Typical signal as recorded by the Timepix detector during helium ion beam imaging. The detector was operated in the energy mode. Larger clusters correspond to helium ions, the smaller ones are due to lighter secondary particles

The sensitive layer of all 5 detectors is in our case made of 300 μm thick crystalline silicon. In the case of the energy-loss detector, this small thickness limits the sensitive WET range of the system to about 1.2 cm [20]. Possible mitigation strategies of this limitation are discussed in “Outlook” section. The sensitive layer of each detector is bump-bonded to the readout chip pixel-by-pixel, as shown schematically in Fig. 2 left. The Timepix detector provides a sensitive area of 14 × 14 mm^2^, divided in square pixels of 55 μm × 55 μm. For each pixel the Timepix ASIC contains the whole electronic chain. The 14-bit digital counter provides a dynamic range of 11,810 counts. In order to minimize the probability of ion scattering in the detectors, we used readout chips thinned down to 100 μm by the Advacam company. For collection of the charge carriers generated by single ions in the detector, a reverse bias voltage was applied. The optimal values of the parameters including the bias voltage, acquisition time and timing frequency were derived in dedicated experimental studies presented in “Choice of the parameters of the radiographic system” section.

The signal created by an ion spreads over several neighboring pixels, forming a so called *cluster*, as it is illustrated in Fig. 2 right. Since our detector is perpendicular to the ion beam, the clusters have typically a round shape. The cluster volume is related to the energy deposition of single ions (see “Settings of the Timepix detectors” section). The cluster size is the number of pixels within one cluster, which exhibit signal above threshold. The determination of the center of mass of each cluster enabled us to reach sub-pixel resolution in the determination of the position in the plane perpendicular to the beam.

The Timepix detector is capable of operation in several modes. We used the “time mode” for tracking, based on the measurement of the arrival time with 100 ns precision. This enabled us to identify hits by one particle in the two pairs of tracking layers in front and behind the phantom. Given by the pixel size and the distance of both detectors in a tracker, each tracker has an angular resolution of at least 0.36°. This leads to a spatial resolution (in planes perpendicular to the beam direction) below 0.3 mm along the whole phantom length.

The “energy mode” was exploited to acquire the information on energy deposition in the detector layer directly behind the phantom. Moreover, clusters measured by the energy deposition detector were used for ion identification based on the previously developed pattern-recognition algorithm, using both cluster volume and cluster size (Gallas et al. 2017).

For the detector readout, one interface was used for the front tracker and one for the rear tracker together with the energy deposition detector. The used readout interface FitPIX (by Advacam s.r.o., Prague, Czech Republic) [39] has the capability of synchronized operation of up to 8 detector layers connected to it [61]. Moreover, several FitPIX interfaces can be operated in synchronization. This capability was one of the key features of our experimental approach, enabling us to associate single outgoing ions to the corresponding incoming ion. The software package Pixet (v.1.4.2, by Advacam s.r.o., Prague, Czech Republic) was used to set the parameters of the detector (see “Choice of the parameters of the radiographic system” section) and to control the readout, data acquisition and recording.

The image acquisition speed was on average 25 frames per second. There were on average about 30 helium ions per frame, corresponding to an average fluence rate of about 150 helium ions/(s mm2). The active imaging time for doses corresponding to diagnostic radiography (350 μGy) was below 11 s (see Fig. 6f). The real imaging time – in this case about 7 min - was dominated by the dead time of the detector.

### Dedicated data analysis method and image formation

With the built radiographic device, we performed experiments at the clinical ion beam therapy facility HIT in order to address its capabilities for imaging structures of relevant sizes (1 mm), as desired in the therapy (see above). We developed an extensive dedicated data analysis method aimed at a maximal exploitation of the measured information for imaging [20]. It comprises cleansing of the raw experimental data and extraction of the information about single ions crossing the imaged object. The method is implemented in a dedicated software package written in MATLAB^3^ and C++. It includes the following steps:

- cleansing of the raw data by excluding light secondary background radiation (electrons and photons)
- identification and removal of detector artifacts due to spatially or temporally incomplete signal readout.
- identification and removal of overlapping signals produced by more than one particle
- homogenization of the detector response by a per-pixel-calibration in terms of energy deposition [31]
- identification of the outgoing ion type (helium or hydrogen) and excluding the hydrogen ions
- tracking of single ions in front and behind the imaged object

The core of the data analysis software is a matching algorithm [21] which enables us to assign single outgoing particle tracks to the corresponding single primary impinging helium ion tracks based on their arrival time. Furthermore, the information measured by the energy detector, which does not carry any time stamp, had to be associated with the measured tracks. To accomplish it, the measured outgoing tracks were extrapolated to the energy detector to find the closest cluster. If within 4 pixels (220 μm), this cluster was considered to come from the same particle.

If the outgoing ion was found to be helium, the measured energy deposition information contributed to the measured image. The imaging plane was positioned in the middle of the phantom, where the inhomogeneity to be imaged is situated. A connection line between the measured incoming and outgoing position of the helium ion on the phantom surface was established. The measured energy deposition was associated to the position where the line crosses the imaging plane [21].

The quality of the images was evaluated quantitatively in terms of CNR and SR. The SR was obtained by the oversampling technique [17, 44]. The values are given in line pairs per millimeter at 10% of the modulation transfer function (MTF) and as full width at half maximum (FWHM) of the line spread function.

### Monte Carlo simulations

In addition to the performed experiments, the whole experimental setup and the beam passing through it were modeled in detail in the Monte Carlo code FLUKA version 2011.2c.3 [6, 16]. FLUKA is the code currently best benchmarked against experimental data in the field of ion beam radiotherapy. The simulations enabled us to optimize the experimental setup and the beam energy before the measurements. This allowed us to save the experimental beamtime at the HIT facility. Moreover, the measured and modeled energy deposition were compared. Calculation of the dose to the imaged phantom was also performed with MC simulations. In the FLUKA simulations, the default parameter set called HADROTHErapy was used. However, there was a need to adjust some of the parameters in order to reach a sufficient accuracy in the simulation of energy loss of ions in the thin detector layers [19]. In particular, the threshold for delta ray production was lowered from the default 0.1 MeV to 0.01 MeV. In this way the possibility that the delta rays escape the thin sensitive volume was accounted for. Moreover, the step length of charged hadrons was forced to stay below 1 μm within the detection layer, which was found as an optimum between the accuracy and computational time.

For modeling of the primary ion beam, pre-generated phase spaces were used [65], which account for the influence of the beam line and the beam nozzle on the ion beam. The detector structure was modeled as far as it was known. The implementation of the bump-bonds and the readout chip was found to be relevant for modeling of the energy deposition of the beam in the detectors, which was found to be influenced by back-scattering effects by about 1% [19]. A complete reconstruction of the final simulated data was performed in accordance to the analysis of the experimental data.

### Studies and experiments

The imaged phantoms consisted of head-sized (160 mm thick) blocks of Polymethyl methacrylate (PMMA). They contained 1-2 mm structures, corresponding to clinically relevant WET variations to be imaged (0.6 and 1.2%, respectively), inserted at different positions in depth [20, 21]. Imaging of the phantoms was performed using therapeutic helium ion beams at HIT. For imaging with helium ion beams, the energy and thus the range of the helium ion beam was chosen so that the beam crosses the entire phantom and the detection system, and the rising part of the Bragg peak was positioned in the active area of the rear detector. In the case of the used head-sized phantom the initial beam energy was 168.3 MeV/u. Single pencil beams with a FWHM of 10.6 mm were found to be sufficient to cover the active area of the detector (2 mm^2^) with a fluence profile of adequate homogeneity. In order to minimize the number of clusters caused by more than one particle (overlapping signals), the applied fluence rate was reduced by about 2-3 orders of magnitude with respect to the lowest fluence rate that is used during clinical treatments. In this way an occupancy below 1% was reached for all detector layers.

For an accurate single particle tracking, a precise alignment of the five detector layers is crucial. Laser system installed in the experimental room was used for manual positioning of the detector, together with a developed support structure. This method allowed us to reach a precision below 1 mm. The precision was further increased by an experimentally determined correction in terms of offsets of single detector layers in the two directions perpendicular to the beam axis (x and y direction) [21]. The first detector behind the phantom, which was positioned in the isocenter, was chosen as reference. The narrowest available helium ion beam with an FWHM = 4.9 mm at the highest energy of 220.5 MeV/u, was used. It was sent through the detection system, while the phantom between the forward and the rear tracker was removed. On each detector layer, the beam position was determined as the point of the maximum fluence. In this way, relative x and y offsets of the detectors 1,2,4 and 5 with respect to the reference detector 3 could be determined. This correction was applied offline, during the data processing.

In the radiography technique developed within this project, energy deposition of single ions in thin (300 μm) silicon layers is the main quantity for forming radiographic images [20, 21]. Therefore, first the capability to measure single ion energy depositions quantitatively was investigated experimentally using a single detector layer operated in energy mode in a free-in-air setting. For this, therapeutic monoenergetic ion beams with a well defined energy and isotope composition over the whole energy range available at HIT were used. Except of helium ions, also protons and carbon ions were investigated in order to extend the LET region up to the values typical for the helium Bragg peak. The cleaned energy deposition spectra were quantitatively compared to FLUKA Monte Carlo simulations (“Settings of the Timepix detectors” section). These experiments were also used for the determination of an optimal value of the bias voltage to be applied to the sensor in order to collect the signal.

The performance and efficiency of the image processing steps (“Dedicated data analysis method and image formation” section) were evaluated on experimental data (“Dedicated data post-processing method” section). The amount of quantities measured on single-ion basis made it possible to construct different imaging modalities: besides deposited energy, also its standard deviation, fluence attenuation, angular distribution of the outgoing ion with respect to the incoming ion, and cluster size. Their suitability and performance for visualization of a 1 mm high air inhomogeneity (step), being the ultimate goal for clinical application, were evaluated quantitatively (“Performance of the helium ion imaging” section).

Radiation-induced changes of the detector response, potentially deteriorating the image performace over time, were investigated using a well defined low LET radiation (a monoenergetic 200 MeV proton beam) [5]. To do so, one of the detectors was exposed to a homogeneous proton fluence increasing in logarithmic steps, up to a cumulative value of 4.64 × 10^11^ protons/cm^2^. The changes of the response during and after the irradiation were accessed by the evaluation of the quality of the energy deposition measurement and the detector response homogeneity.

## Results

### Choice of the parameters of the radiographic system

The versatile Timepix detection technology provides a high level of freedom concerning the detection system assembly and the setting of the data acquisition parameters. Here we detail our considerations and studies leading to the selection of the final detection parameters.

#### Concept of the detection system

The main radiographic information, the energy deposition, was measured by the energy deposition layer. This layer also provides information on ion identification. Since the heavy bump-bonds have the potential to deteriorate the image by an artificial increase of the measured energy spread, the energy deposition detector was positioned in front of the rear tracker [21]. This non-standard placement enabled us to minimize the spread of the energy loss measured.

The implementation of a tracker composed of both the front and the rear part enabled us to investigate the contribution of the information on incoming and outgoing particle directions to the image quality independently. Moreover, such a system is applicable also for proton based imaging, where including of the position and directional information in front and behind the object is necessary (see “Background” section).

With an increasing number of layers per tracker, the amount of scattering of the imaging ion beam is also increased. This is particularly pronounced for the rear tracker, since the outgoing ions are close to the end of their range, and thus very slow. Therefore it is desired to minimize the amount of material per tracker.

For the determination of both particle hit coordinates in the plane transversal to the beam, one pixelated Timepix layer is sufficient, in contrast to the two or more strip detector layers used standardly. Since the detection efficiency of Timepix for therapeutic ions approaches 100% per layer, two layers in the front tracker and another two layers in the rear tracker were used to determine the entrance and exit position and direction of each ion. Moreover, readout chips thinned down to 100 um were used.

The small thickness of the used components, including the cooling, enabled us to position the closest layers of the system less than 4 cm from the imaged phantom [21]. The alignment of the whole detection system was performed using the laser positioning system (see “Studies and Experiments” section). Residual lateral misalignment were determined (see “Studies and Experiments” section), yielding offsets below 4 pixels in both directions perpendicular to the beam axis, corresponding to 220 μm. The measured offset values were applied to correct the measured cluster positions offline. This procedure resulted into an alignment better than 1 pixel (55 μm) for all the five detector layers with respect to each other.

#### Settings of the Timepix detectors

The detector layer used for energy deposition measurement was operated in the energy mode, while the layers for tracking were operated in the time mode. For the whole system the acquisition time and the bias voltage were optimized [19].

The energy deposition in the energy detector for the reported measurement was typically 5.1 ± 0.6 MeV [20]. In order to digitize the corresponding signal, 260 ± 40 μs are needed. The frame duration (acquisition time) of 1 ms was determined as an optimum between the amount of fully digitized particle signals and the amount of overshoot signals.

**Fig. 3:**
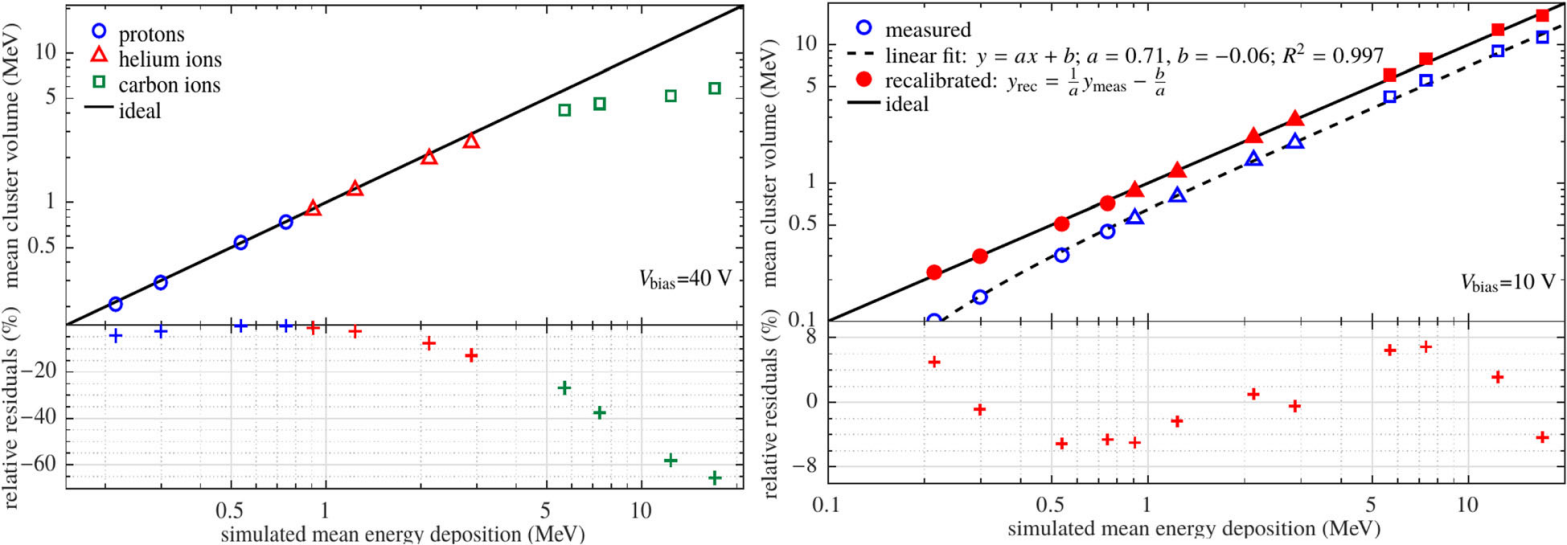
Correlation between the measured mean cluster volume and the energy deposition in a 300 μm thick silicon layer of the detector as simulated by FLUKA. Different energy depositions levels were reached using monoenergetic protons, helium ions and carbon ions. Left: V_bias_ = 40 V. Right: V_bias_ = 10 V. Measured data are shown in blue, data after the developed recalibration procedure are in red. The lower plots show the residual differences between the measurement and simulation. Figures are from [19]

The impact of bias voltage on the measured signal in the energy deposition detector were evaluated for a fully and for a partially depleted sensor [19]. At 40 V the sensor is fully depleted. In this case the measured cluster volume is in agreement with Monte Carlo simulations of the energy deposition within 7.7% for energy depositions below 2 MeV, as illustrated in Fig. 3 left. However, for higher energy depositions the signal suffers increasingly from a nonlinearity of the detector response, due to improper digitization. Indeed, at typical energy depositions for our helium radiography around 5 MeV, the quenching exceeded 20%. In the perspective of helium ion radiography, this leads to a decrease in the image contrast.

In the case of a bias voltage of 10 V, the sensitive layer is not completely depleted. Consequently, the sensitive volume is reduced. Therefore, just a fraction of the generated charge is collected at the readout electrodes. For this lower signal the response of the detector was found to be linear in good approximation (see Fig. 3 right). This leads to an improved image contrast in comparison to 40 V. However, due to the partial depletion, the measured energy deposition value cannot be compared to Monte Carlo predictions in a straight forward way. To account for this effect, we developed an iterative recalibration procedure of the detector response [19]. The measured data after recalibration agree with the Monte Carlo prediction within 7% over the whole investigated region of energy depositions from about 0.2 to 17 MeV in 300 μm silicon (or 0.72 - 56.63 MeV/mm).

The bias voltage of 10 V is also beneficial for the tracking accuracy. Higher voltages lead to a decreased cluster size, yielding a decreased precision in the determination of the particle impact. In addition, a superior homogeneity of the detector response to monoenergetic ion beams was found at 10 V [5]. These findings led to the conclusion to operate the detector at a bias voltage of 10 V for acquisition of the radiographies.

### Radiation-induced changes of the detector response

**Fig. 4:**
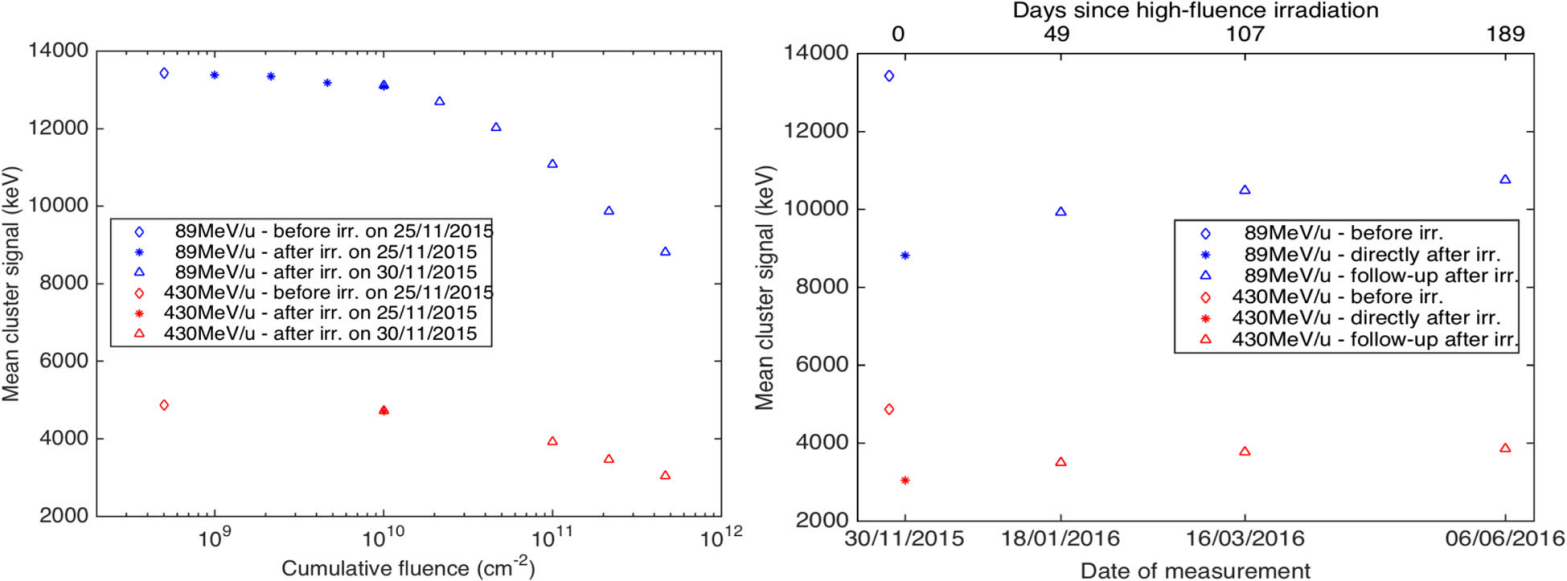
Evaluation of the detector response changes after irradiation by a cumulative fluence of 4.64 × 10^11^ protons/cm^2^ with an energy of 200 MeV. Changes of the mean cluster signal (equal to cluster volume) during (left) and after (right) the high fluence irradiation are shown. The changes are depicted for both the highest (430 MeV) and the lowest (89 MeV) monoenergetic carbon ion beams available for therapeutic treatments at the HIT facility. Figures are from [5]

The short time stability of the detector response (several hours) in terms of energy deposition was found to be within 0.9% for energy depositions between 0.2 and 17 MeV [5]. The stability of the detector response with respect to a high dose irradiation were studied using fluences of 200 MeV protons with fluences of up to 4.64 × 10^11^ p/cm^2^, corresponding to a total dose-to-water of about 330 Gy [5]. The changes of the response were measured for monoenergetic proton, helium and carbon ion beams with energy depositions between 0.2 and 17 MeV. The closest energy deposition to the radiographic helium ions had the carbon ion beam of the highest energy – 430 MeV/u. The results in Fig. 4 left show that for this energy deposition the changes are minor (within 3%) up to fluences of 10^10^ p/cm^2^, which corresponds to about 7 Gy. After 330 Gy the measured cluster volume decreased by about 30-40% with respect to the status before the irradiation started.

Furthermore, an improvement in the homogeneity of the response following the irradiation was observed. The changes of the measured deposited energy spectra and the detector homogeneity were partially reversed in the months after the irradiation as shown in Fig. 4 right.

### Dedicated data post-processing method

Besides the “true” helium signal, radiation background and signals degraded due to different effects and detector artifacts were found among the measured signals [20]. We successfully assigned the different signal components to the respective causative mechanisms. This knowledge was the basis of the developed data post-processing procedure for cleansing of the measured data (see “Dedicated data analysis method and image formation” section).

The main measured radiographic quantity was the cluster volume, which is a linear function of energy deposition of the ions in the detector in the LET range of the primary and secondary particles present, as shown in Fig. 3 right. Although the quantitative measurement of energy deposition is not mandatory for radiography, an understanding of the effects influencing the measured cluster volume of the helium ion signal is needed for the development of a correct and efficient data processing. In a detailed analysis of the measured signal we found that several effects have the potential to influence the quality of the measured cluster volume significantly [20]:

- Although the intensity of the beam was kept at low values (see “Studies and Experiments” section), there is a non-zero probability for signals due to two or more overlapping clusters at the energy deposition detector. They were identified by multiple maxima within a single cluster and excluded from further consideration.
- To suppress overlapping clusters in the tracking detectors, a maximum allowed deviation on the measured arrival time in pixels of one cluster was used. This cut was found to suppress the so called “overshoot clusters”, too. This kind of detector artifact arises due to the oscillation of the readout electronics when the deposited energy in a single pixel is above approximately 1 MeV [25].
- The energy deposition of clusters cropped temporally is digitized only partially. Such cluster might occur at the beginning or at the end of the acquisition time within a frame. Therefore, clusters produced by particles arriving too early or too late to be fully digitized were not considered in the further data analysis. However, the signal from the energy deposition does not contain any time information. The arrival time information recorded for the same particle by the tracking detectors operated in the time mode was used to determine the time of the particle impact on the energy deposition detector.
- Although an absolute energy deposition measurement is not needed for ion radiographies, a calibration of all 65,536 pixels in terms of deposited energy using low energy X-rays [31] has shown an improvement in the homogeneity of the detector response.

The purification of the signal by excluding the radiation background (photons, electrons and activation products) and artefacts caused by the detection technique (overshoot clusters and overlapping clusters) pass only ion-caused clusters, which represent about 1/3 of all registered clusters. About 65% of them can be matched with signals on all the four tracking detectors, which are induced by the same particle. This fraction would further increase with larger detector areas. The subsequent removal of temporally cropped clusters with a deteriorated energy deposition information, and rejection of hydrogen ions, decrease the number of usable events by 12-13% each.

**Fig. 5:**
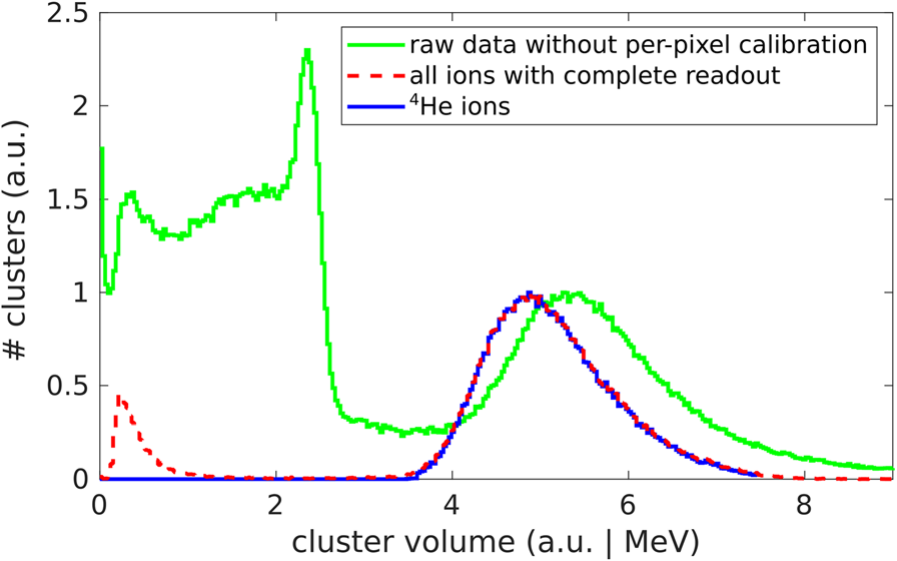
The effect of the data processing steps illustrated on the cluster volume spectra of helium ions measured within a radiography

The effects of the above data processing steps on the spectra of energy deposition measured for a helium radiography is illustrated in Fig. 5. The cleaning of the raw signal spectrum includes a removal of photon and electron clusters, artifacts due to temporally or spatially cropped cluster volumes and overlapping clusters. After the application of signal calibration, a clean ion spectra remains. This contains both helium and hydrogen ions. The ion identification procedure restricts the signal to helium ions only.

### Performance of the helium ion imaging

The performance of the radiographic system we designed and built (see Fig. 1) was investigated experimentally for helium ion imaging [21]. We aimed to resolve a clinically desired thickness difference in the beam direction of 1 mm, corresponding to a relative WET difference of 0.6% in the used head-sized PMMA phantom. This inhomogeneity was modeled by a 1 mm air slab, positioned in the center of the phantom and thus at the maximal distance to both tracking modules.

**Fig. 6:**
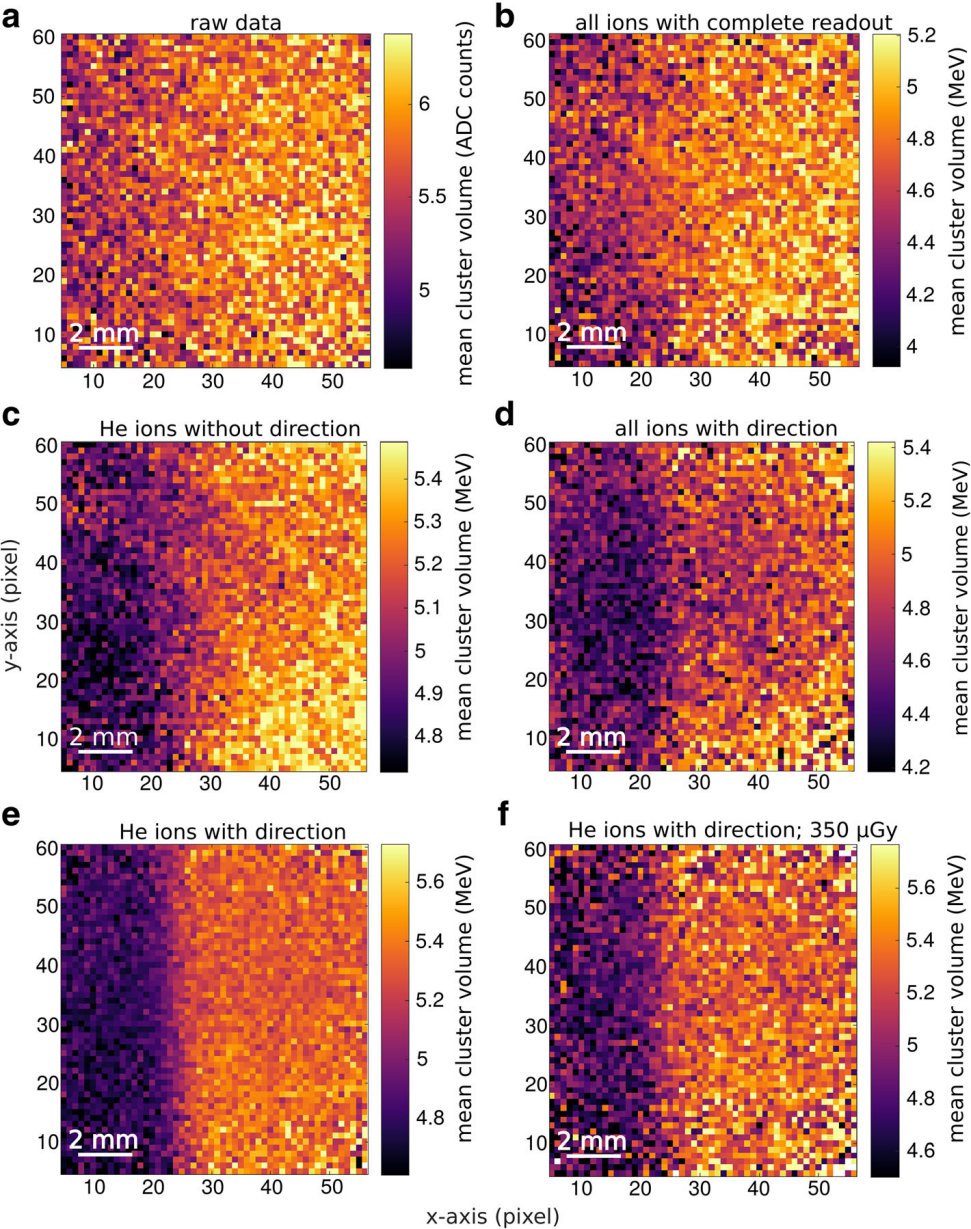
Evaluation of the spatial resolution of helium ion radiography for a measured radiograph depicting an air inhomogeneity 1 mm thick (in the beam direction) in an otherwise homogeneous PMMA phantom of 161 mm thickness. The inhomogeneity was positioned in the middle of the phantom, where the lowest theoretical resolution is expected. The panels **a**) to **f**) show the data after single data processing steps. Panel **e**) shows the radiograph after the complete data processing at the dose level of 1.44 mGy, while panel **f**) shows the image quality at the dose level of diagnostic X-ray radiographies (350 uGy). The pixel size is 220 μm × 220 μm

The resulting images are structured in 220 × 220 μm^2^ pixels, which are substantially finer than the clinically desired SR of 1 mm. Panel a) in Fig. 6 shows the cluster volume distribution over the detector area as measured, without any data processing steps applied. It illustrates that without further data processing the detection method is not capable to visualize the aimed inhomogeneity. In the same Figure, panels b)-e) illustrate the impact of single data processing steps (see “Dedicated data analysis method and image formation” section). The removal of detector artifacts, shown in panel b), increases the CNR by a factor of approximately 1.4. Panel c) shows the effect of excluding light secondary radiation (hydrogen ions) from the image generation, which increases the CNR by a further factor of 2.1. Panel d) shows the effect of considering of the measured direction of the ions in front and behind the imaged phantom, which improves the CNR by an additional factor of 1.6. In this image the measured cluster volume was positioned at the point where a line connecting the entering and exit point of the ion traversing the imaged phantom crossed the transversal plane situated in the middle of the phantom, where the inhomogeneity was positioned. The entering and exit point were determined by extrapolations of the measured directions to the phantom surface. The consideration of the ion direction improves the SR by a factor of 2.5. Panel e) shows the effect of a simultaneous consideration of the measured ion direction and selection of helium ions only. The performance of the imaging for a clinical dose for head radiography (350 μGy [20, 56]) is depicted in panel f). The edge is still clearly visible.

**Fig. 7:**
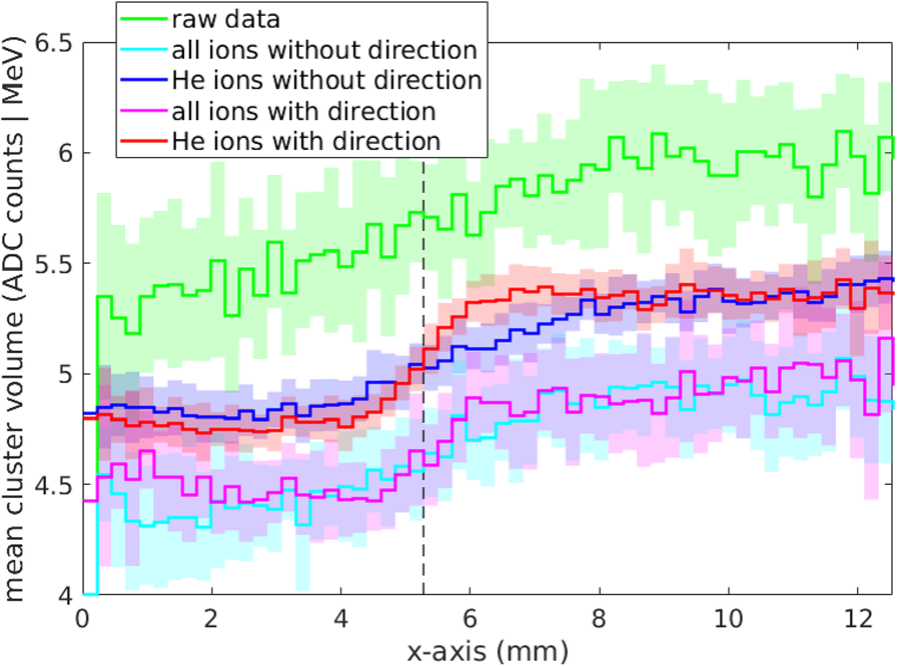
Measured profiles of the helium radiographs of a 1 mm air inhomogeheity (see Fig. 6) after single data processing steps. The profiles were averaged over 20 superpixels along the y-axis, corresponding to 4.4 mm

To facilitate a quantitative comparison of the image quality, Fig. 7 depicts line profiles along the x-axis of the panels a)-e) of Fig. 6. The initially invisible edge becomes clearly visible after passing all the steps of the data processing.

**Fig. 8:**
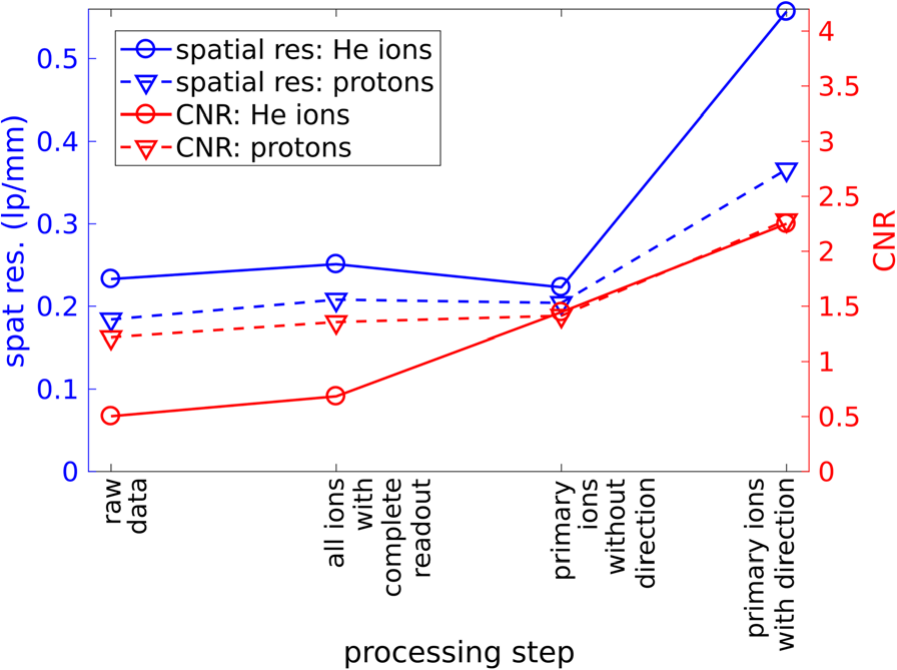
Measured CNR and spatial resolution for a 1 mm air inhomogneity as a function of the data processing step (see Fig. 6) is shown for protons and helium ions. The dose level was 450 uGy

Figure 8 shows quantitative changes in the CNR and SR due to the single data processing steps. In total, CNR increased by a factor of 4.5 in comparison to the raw data without the dedicated post-processing. The final CNR reached the value of 2.3 at the investigated position for a dose of 450 uGy. For protons a comparable value was reached at the same dose.

As expected, the SR increases in particular due to the consideration of the measured directions of the ions in front and behind the imaged phantom. A factor greater than 2.5 was found for helium, and a factor of 1.8 for protons. The helium radiography exhibits spatial resolution of 0.56 ± 0.04 lp/mm at the MTF_10%_. Thus it is 50% higher than the spatial resolution of 0.37 ± 0.02 lp/mm, which was measured for protons in the exactly same setting.

## Discussion

Within the development of the precise imaging method, we optimized the detector design, and established a dedicated data acquisition and evaluation procedure. The performance of the system was demonstrated experimentally [21]. The developed Monte Carlo simulation of the whole detection system helped us to understand the observed effects and to optimize the method. A complete reconstruction of the simulated data was implemented in accordance to the analysis of the experimental data.

### The imaging method

#### Detection system, its optimization and characterization

The designed and built radiation detection system was optimized for single ion radiography. The chosen radiation detection technology Timepix exhibits sufficient geometrical segmentation and temporal resolution to register single therapeutic ions. Moreover, it provides a high level of freedom concerning the building of the system, as well as the data acquisition parameters.

The imaging method is based on the measurement of the energy deposition in the rising part of the Bragg curve [20]. The energy detector is complemented by a tracker composed of the front and rear part. Therefore, the system is applicable for both helium and proton imaging, and thus it enables their direct comparison [21].

While the majority of the published ion imaging systems are, at least partially, based on scintillation detectors [51], fully pixelized semiconductor based detection systems are scarce [48]. Although the electronics for pixelized detectors is significantly more complex than for 1D detectors, this approach has several advantages. While scintillating fibers or silicon strip detectors provide only one coordinate of the particle hit per layer, a tracker based on pixelated detectors provides both coordinates of a particle hit in each layer. The WET of a single used Timepix layer with a thinned readout is about 1 mm, which minimizes the scattering of the ions in the imaging system. Moreover, the pixel technology allows to lower the occupancy of the tracker and enables an improved disentangling of situations where multiple particles are detected in the same time window, e.g. for multiple nuclear fragments originating from the same primary ion.

Our concept is unique in using a single technology for the measurement of the energy deposition, tracking and ion identification [21]. This allows e.g. a straight forward investigation of different order of tracking and energy deposition modules, which is difficult, and often even impossible, with the existing detection systems. The developed detector alignment procedure enables us to reach subpixel accuracy of the position of the detector layers with respect to each other.

Optimal settings of the detection system, like the acquisition time duration and bias voltage, were found in dedicated studies by maximizing the CNR and SR [5, 19]. For the measurement of the energy deposition, a fully depleted detector was found to produce too high signals that exceed the linear regime of the detector. A partially depleted sensor provides a lower signal, that leads to a larger effective dynamic range and thus an improved image contrast.

The unique positioning of the rear tracker behind the energy deposition detector enabled us to minimize the deterioration of the energy deposition information by the interactions of the ions with the tracker. A comparison with MC simulations have shown that the accuracy of the measured energy deposition, with the developed recalibration procedure, is below 7% for energy depositions between 0.2 and 17 MeV in 300 μm silicon [19]. A potential for a further increase in accuracy was found in the systematic trend of the found differences.

In a study about radiation hardness, we observed that for the investigated detector there can be relevant and time-dependent changes of the response due to radiation above 7 Gy [5]. This shows that a monitoring of the detector response, and performing a recalibration if necessary, is important for high quality radiographies.

#### Data processing method

A dedicated data processing method was developed. It includes an identification and removal of radiation background and detector artifacts, homogenization of the detector response, single ion identification and tracking [20]. Finally, all signals in the 5 detectors, which originate from a single particle, were matched [21]. The improvement of the images by different data processing steps was evaluated in detail. In the energy deposition spectra we successfully identified sources of background – secondary electron and photon radiation, image artifacts due to temporally and spatially incomplete signal readout, overlapping signals and overshoot signals. Their removal improved the CNR by 40% in comparison to the raw data.

When ions heavier than protons are used, a challenge is represented by the nuclear fragments of the primary ions which have a different energy deposition than the primary ions. This leads to an increased image noise and thus limits the WET resolution in the direction along the beam. That issue was addressed by including an ion identification capability, which is based on pattern recognition of the signal measured in the energy detector (Gallas et al. 2017). The removal of identified hydrogen ions improved the CNR by further 110%.

Finally, the consideration of the measured entering and exit position of single ions improved the CNR by additional 60%. The total improvement of the image quality in terms of CNR achieved by the developed image processing method reached 350%.

As expected, the largest improvement of the SR was gained by the consideration of the entrance and exit position of single ions. It was found to be as high as 150%.

#### Performance of the system for helium and proton imaging

The criteria for a clinically applicable ion imaging system include SR, density / thickness resolution for clinically applicable doses, the size of the field of view, imaging time, image reconstruction speed, radiation hardness, issues of patient safety and dimensions of the device making it feasible for implementation in the treatment rooms.

With helium ions the CNR, which quantifies the resolution in tissue thickness or density, was found to be high enough to visualize the 1 mm step (or 0.6% WET difference) in a head-sized phantom at a diagnostic dose of approximately 350 μGy. For protons the CNR was comparable at the same dose level. In case of carbon ions the image quality was found to be limited by the low number of carbon ions per pixel [21].

Publications on helium ion beam imaging, which we could compare our results to, are rare. Approaches based on passive detectors [7] have low applicability for the current high throughput facilities. The reported active systems for helium imaging are mainly tomographic. The first system based on scintillating paddles and an MWPC tracker was published already in 1975 [13]. It was capable to visualize a density difference below 2% in a head sized phantom at a clinically feasible dose. Since its advantages over a clinical CT (status at that time) were demonstrated, it was even approved for a trial with humans.

In [45] another helium CT system was presented. It was based on a plastic scintillator calorimeter and a scintillating fiber tracker. A WET resolution of 1.5% was found for cylindrical phantoms significantly smaller than an adult head. The imaging dose is not explicitly given. That system was tested also for carbon and neon ion imaging. A helium imaging study with a system designed for proton imaging is reported in [67]. The system consists of two silicon strip trackers and an energy/range detector based on a plastic scintillator. The relative stopping power accuracy was found to be 2.5% or better in a helium CT of the used phantom.

For comparison, in proton imaging [57] reports a WET resolution of 0.6 mm for 100 protons per pixel for a proton CT system evaluated with head sized phantoms. [3] reports a WET resolution of 3.05 ± 0.3 mm per proton at the maximal thickness of the cylindrical phantom of 20 cm WET. A range resolution of 8.4% with a systematic deviation from the expected range of about the same size is reported in [48] for proton imaging with a digital tracking calorimeter. In that work the deposited energy was determined indirectly from the cluster size.

The high CNR found in the present study makes the developed method promising for a direct visualization of targets with a small WET difference to the surrounding tissue, at clinically feasible doses. In this way the use of fiducial markers, whose placement is invasive, could be avoided.

Additional contrasts due to fluence attenuation, cluster size, particle angle and spread of the measured energy loss in a pixel were investigated. All of them were found to be lower than the contrast due to energy deposition, which is used in the final method.

With the novel ion imaging system a spatial resolution of 0.56 ± 0.04 lp/mm at the MTF_10%_, was reached for imaging of a 1 mm step in a head-sized PMMA phantom with helium ions. As expected, due to the increased multiple Coulomb scattering, the SR for protons was found to be lower – only 0.37 ± 0.02 lp/mm. These values were obtained for the inhomogeneity position in the middle of the phantom, which has the maximal distance from both tracker parts. The superior spatial resolution of the helium radiography was found at a comparable thickness resolution (CNR) and imaging dose [21]. Possible further improvements of the spatial resolution with this system, in particular the performance of different image reconstruction algorithms, were studied in our further research [21]. Spatial resolution in terms of MTF_10%_ was found to be 0.61 lp/mm for helium and 0.34 lp/mm for protons in [67]. Due to the different sizes of the phantoms (10 cm vs. 18.6 cm WET), these values are not directly comparable to our findings.

For comparison, to proton CT systems evaluated with head-sized phantoms, [57] reports SR of 3.53 mm FWHM for the worst-case scenario. Plautz et al. [49] found the radial SR to be 0.511 ± 0.061 lp/mm at MTF_10%_ at the maximal phantom thickness of 20 cm WET.

The system also exhibits further properties important for a clinical application. In contrast to systems with trackers based on multiwire proportional chambers, it does not require any high voltage and gas filling. This increases the patient safety and keeps the size of the system small. With the weight below 0.5 kg, the current prototype is light enough to be mounted on gantries. Its flexibility is important with respect to further developments.

The imaging time was largely dominated by the dead time of the detector (see “The Timepix detectors” section). However, there are technologies to overcome this in the near future (see “Outlook” section).

With this kind of system, interfractional imaging of the patient could be performed directly before the treatment start. While the patient is in the treatment position, the detectors could be positioned in front and behind him. After the imaging, the detectors would be removed in order to not impair the quality of the treatment beam. For intrafractional imaging the treatment would have to be paused during the imaging, since for both the ion beam is needed, however with different energies. In contrast to ion computed imaging, no rotation of the beam or the patient is needed for ion radiography, what makes it faster and less complicated, and thus more suitable for first clinical applications.

### Outlook

The sensitive WET range at which thickness differences can be detected with high resolution is currently about 1.2 cm (see “The Timepix detectors” section). This is due to the width of the rising part of the Bragg curve. There are several options to be investigated to overcome this limitation. An use of a spread-out Bragg peak with several energies like in [70] would lead to an increased dose to the patient. An implementation of multiple layers interlayed with absorbers would increase the cost and the number of channels proportionally. Another option would be to use pencil beams with different energies for different lateral regions of the patient. The pencil beams would have to be narrow enough to cover areas with WET variations below 1 cm.

In contrast to broad beam imaging, with scanned ion beams the size of the imaging field can be precisely adjusted to the size of the target area. In this way the dose to healthy tissue can be minimized. The remaining imaging dose can be partly accounted for in the treatment planning.

The dead time of the Timepix detector (see “The Timepix detectors and Performance of the system for helium and proton imaging” sections) will not remain to be a limitation in the future. The Timepix 3 detector [50], a successor of Timepix, provides a faster readout and a dead time free operation. With this detector the imaging is expected to correspond to the active imaging time. Its duration will depend on the parallelity of the readout of the clinically sized detection system.

The limitation concerning the small field of view (2 cm^2^) of the current detector system prototype is also not fundamental. Large-area Timepix based detectors are already commercially available. Multilayered detectors with low material budget and comprising sufficient cooling are to be developed. The price per sensitive area is certainly higher for a hybrid pixelated detector than for silicon strip detectors and calorimeters with several channels. However, the price of a high resolution radiographic device has to be considered in relation to the price of a highly precise ion beam therapy facility. Following the past trend, the prices of semiconductor detectors can be expected to further decrease in the future. Due to the planned upgrades of the large hadron collider at CERN, there is a vivid development of new pixelized semiconductor radiation detectors. Also therefore, the technology of pixelated semiconductor detectors has a positive future perspective concerning further developments in terms of speed and functionalities [8]. E.g., besides the increased speed of data acquisition, the Timepix 3 detector has the capability to measure the energy deposition and the time of arrival in each pixel simultaneously. This opens the possibility to reduce the number of layers, and therefore to further decrease the influence of ion scattering. Pixelated detectors are also becoming commercially available, which boosts their investigation for possible application in medical physics.

## Conclusions

Due to the high conformation of the dose to the target, ion radiotherapy would profit even more from enhanced image guidance than the standard radiotherapy with photons. A decrease of the uncertainty of the target position knowledge can be directly translated to lower dose to the healthy tissue. Therefore, imaging methods with high sensitivity to minor areal density changes and high resolution in the plane perpendicular to the beam direction usable for the monitoring of the target and for patient positioning are of a major interest.

Ion radiation provides potentially high image contrast due to the steepness of the Bragg curve. However, the development of dedicated radiation detection systems is still in an experimental phase. For future monitoring of the internal target position, our aim was to image a WET difference of 1 mm at clinically acceptable dose levels with a position accuracy of about 1 mm in the plane perpendicular to the ion beam.

Helium ion beams were chosen as imaging radiation, as the multiple Coulomb scattering, which limits the achievable SR, is lower for helium ions in comparison to protons. At the same time the radiation damage to the healthy cells is lower than for carbon ions.

We report on the development of a dedicated helium ion radiography method, including the design and building of the imaging system. It is composed of an energy loss detector complemented by a front and a rear tracker for improving of the spatial resolution. A dedicated data acquisition procedure and information postprocessing were established.

The performance of the method was evaluated experimentally at the ion beam therapy facility HIT in Germany. Both SR and WET resolution of the images reached at diagnostic dose levels were assessed in detail.

The method enables a clear visualization of an 0.1 g/cm^2^ (or 0.6%) WET-difference at a diagnostic dose level. To reach this performance, a unique method for single ion identification was used to avoid degradation of the images due to the inherent contamination of the outgoing beam with light secondary fragments (hydrogen). At a comparable CNR and dose, helium radiographs exhibited 50% higher SR in the middle of the phantom than proton radiographs. Further improvement of the performance can be reached by the recent algorithms for prediction of the most probable path of the ion in the imaged object.

The demonstrated high performance of the developed helium ion beam radiography method has a high potential for on-couch imaging of even small geometrical changes in the patient.

## Abbreviations

CNR: Contrast-to-noise ratio
FWHM: Full width at half maximum
HIT: Heidelberg Ion-Beam Therapy Center
LET: Linear energy transfer
MTF: Modulation transfer function
MWPC: Multiwire proportional chamber
PMMA: Polymethyl methacrylate
PSI: Paul Scherrer Institute
SR: Spatial resolution
WET: Water equivalent thickness

## Footnotes

1 In this paper we use the term ion radiography for planar transmission imaging, which results into 2D images in the plane perpendicular to the beam. The term ion computed tomography is used for volumetric imaging, leading to 3D images. Smartly performed radiographies might provide the essential information of an ion computed tomography at reduced patient dose, imaging time, technical complexity and cost [14, 57].

2 The detectors, together with the readout interface FitPIX and the Pixet software package were purchased from the company Advacam s.r.o., U Pergamenky 12, 170 00 Praha 7, Czech Republic.

3 MATLAB. Version 9.2.0.556344 (R2017a). The MathWorks, Inc., Natick, Massachusetts, USA.
